# Involvement of Kindlin-1 in cutaneous squamous cell carcinoma

**DOI:** 10.1038/s41389-024-00526-1

**Published:** 2024-07-09

**Authors:** Giovana Carrasco, Ifigeneia Stavrou, Mairi Treanor-Taylor, Henry Beetham, Martin Lee, Roza Masalmeh, Artur Carreras-Soldevila, David Hardman, Miguel O. Bernabeu, Alex von Kriegsheim, Gareth J. Inman, Adam Byron, Valerie G. Brunton

**Affiliations:** 1 Edinburgh Cancer Research, Institute of Genetics and Cancer, University of Edinburgh, Edinburgh, EH4 2XR UK; 2 CRUK Scotland Institute, Glasgow, G61 1BD UK; 3 Centre for Medical Informatics, Usher Institute, University of Edinburgh, Edinburgh, EH16 4UX UK; 4 The Bayes Centre, University of Edinburgh, Edinburgh, EH8 9BT UK; 5 School of Cancer Sciences, University of Glasgow, Glasgow, G61 1QH UK; 6 Division of Molecular and Cellular Function, School of Biological Sciences, Faculty of Biology, Medicine and Health, University of Manchester, Manchester Academic Health Science Centre, Manchester, M13 9PT UK

**Keywords:** Squamous cell carcinoma, Cancer genetics

## Abstract

Kindler syndrome (KS) is a rare genodermatosis resulting from loss-of-function mutations in *FERMT1*, the gene that encodes Kindlin-1. KS patients have a high propensity to develop aggressive and metastatic cutaneous squamous cell carcinoma (cSCC). Here we show in non-KS-associated patients that elevation of *FERMT1* expression is increased in actinic keratoses compared to normal skin, with a further increase in cSCC supporting a pro-tumorigenic role in this population. In contrast, we show that loss of Kindlin-1 leads to increased SCC tumor growth in vivo and in 3D spheroids, which was associated with the development of a hypoxic tumor environment and increased glycolysis. The metalloproteinase *Mmp13* was upregulated in Kindlin-1-depleted tumors, and increased expression of MMP13 was responsible for driving increased invasion of the Kindlin-1-depleted SCC cells. These results provide evidence that Kindlin-1 loss in SCC can promote invasion through the upregulation of MMP13, and offer novel insights into how Kindlin-1 loss leads to the development of a hypoxic environment that is permissive for tumor growth.

## Introduction

Kindlin-1 is a four-point-one, ezrin, radixin, moesin (FERM) domain-containing focal adhesion adaptor protein that binds to β-integrin cytoplasmic tails and is required for integrin activation [1, 2]. As well as regulation of integrin-dependent processes such as cell adhesion and migration, over-expression of Kindlin-1 has been reported in various tumor types, and in some cases such as colorectal and breast cancer, this has been linked to poor patient outcomes [3–9]. However, in esophageal cancer and non-small cell lung cancer, high levels of Kindlin-1 are linked to more differentiated and less aggressive tumor phenotypes, with ectopic expression of Kindlin-1 in non-small cell lung cancer cell lines leading to reduced tumor growth [10, 11]. In addition, loss of Kindlin-1 in patients with Kindler Syndrome (KS) promotes tumorigenesis, with KS patients having a propensity to develop highly aggressive cutaneous squamous cell carcinoma (cSCC) [12–14]. KS is an autosomal recessive genodermatosis resulting from loss-of-function mutations in *FERMT1*, the gene that encodes Kindlin-1 leading to skin atrophy, blistering, photosensitivity, hyper or hypo-pigmentation, increased light sensitivity as well as a high incidence of cSCC [15]. Although numbers are small, a cohort study of 62 patients revealed that 70% of KS patients over 45 years old had developed cSCC, with a further study reporting cSCC development in 13/91 KS patients, with many developing multiple tumors [12, 13]. Little is known about the mechanisms that predispose KS patients to cSCC, although studies in mice have defined a tumor suppressor role for Kindlin-1 in the skin involving control of TGF-β-mediated growth-suppressing signals in cutaneous epithelial stem cells [16]. Studies in clinical samples support a role for TGF-β signaling and stromal fibroblasts in promoting a pro-tumorigenic environment in KS patients [17, 18], while links to UV exposure and inflammation have also been made [12], with reports of upregulation of proinflammatory cytokines in Kindlin-1-deficient keratinocytes [18–20]. In contrast, here we show in non-KS-associated cSCC that *FERMT1* is increased in pre-neoplastic actinic keratoses compared to normal skin, with further increases seen in cSCC, supporting a pro-tumorigenic role for Kindlin-1. This suggests that the increased propensity for cSCC in KS patients may result from the environmental changes in the skin of KS patients.

In addition to the high propensity of cSCC development in KS patients, a number of groups reported that KS patients also developed highly aggressive metastatic disease, that required amputation in some cases [21–24]. However, nothing is known about how the loss of Kindlin-1 in KS patients drives this aggressive and metastatic behavior of cSCC. Here we show that loss of Kindlin-1 in cSCC cell models results in a more invasive phenotype that is driven by increased expression of matrix metalloproteinase (MMP)13. In addition, loss of Kindlin-1 leads to increased proliferation in 3D and increased tumor growth in mice. This is associated with the induction of a more hypoxic environment and increased glycolysis.

## Results

### *FERMT1* expression in normal skin and cSCC

**Fig. 1:**
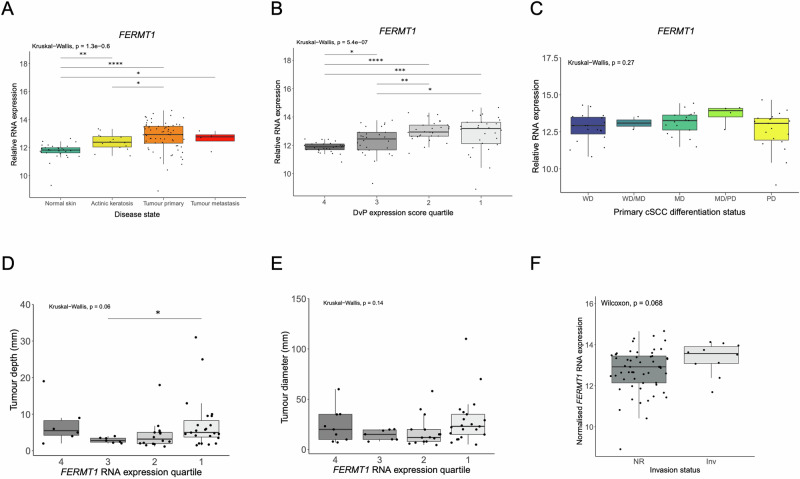
*FERMT1* expression in sporadic human cSCC. Analysis of bulk RNAseq data indicates that *FERMT1* levels increase with disease state (**A**) and DvP quartile (**B**). *FERMT1* RNA levels do not significantly correlate with primary tumour differentiation status (**C**) (WD well differentiated, MD moderately differentiated, PD poorly differentiated). *FERMT1* expression levels do not correlate with primary tumour depth (**D**) or diameter (**E**). *FERMT1* expression levels are not significantly altered in tumours with annotated perineural or lymphovascular invasion (InV *n* = 7) when compared to those with these parameters not recorded on the diagnostic pathology records (NR, *n* = 59) (**F**). Boxplots are annotated by two-sided Wilcoxon rank-sum test or Kruksal-Wallis test as indicated with *P* values <= 0.05 indicating a significant difference between sample groups. *P*-values are not adjusted for multiple testing. The Boxes visualize the interquartile range (IQR) and median, while whiskers show largest and smallest values within 1.5* IQR from upper and lower quartiles. Data beyond the whiskers are deemed outliers.

cBioPortal analysis of the TCGA pan-cancer cohort revealed that *FERMT1* is infrequently mutationally altered (overall 1.2% mutation frequency) in most tumor types (Supplementary Fig. 1A). There are no prominent hot spot mutations with missense mutations present throughout the coding region of *FERMT1* (Supplementary Fig. 1B). Similarly, *FERMT1* mutations have only been observed in 2/83 curated cSCC samples [25]. TCGA analysis of *FERMT1* RNA expression levels revealed overexpression compared to normal samples (log RNA Seq Vs RSEM, Z-score threshold +/− 2.0) in lung, colorectal cholangiocarcinoma and hepatocellular carcinoma suggestive of potential oncogenic roles in these tumor types whilst a preponderance of downregulation of expression is observed in thyroid, kidney, breast, endometrial and prostate tumors indicating potential tumor suppressor roles in these tumor types (Supplementary Fig. 1C). As there are no reports of Kindlin-1 in human cSCC, we analyzed *FERMT1* expression from bulk RNA-Seq data in 110 treatment-naïve patient samples representing normal skin-exposed skin/perilesional (*n* = 26), pre-malignant actinic keratoses (AK) (*n* = 14), primary (*n* = 66) and metastatic cSCC (*n* = 4) [26]. *FERMT1* expression was readily detectable in normal sun exposed skin and we observed a step wise increase in *FERMT1* expression in AK compared to normal, and between primary tumors and AK (Fig. 1A), indicating a potential role in promoting premalignant cell growth and in the transition from AK to cSCC. We have recently shown that cSCC progresses along a disease continuum involving the suppression of epidermal differentiation and induction of progenitor-like gene expression which can be represented as a Differentiation-versus-Progenitor (DvP) score [26]. Analysis of *FERMT1* expression in the bulk RNAseq samples ranked from most differentiated (DvP signature score quartile 4) to most progenitor-like (DvP quartile 1) revealed significant increases in *FERMT1* between quartile 4 to quartile 3 and again between quartile 3 to quartile 2 suggestive of a potential role of Kindlin-1 in de-differentiation and acquisition of a more progenitor-like state (Fig. 1B). To address whether the increased levels of *FERMT1* in less well-differentiated tumors may reflect increased expression in basal keratinocytes, *FERMT1* expression was analyzed in the different cell populations identified in human cSCC that have been shown to reflect the differentiation state of keratinocytes in normal skin [27]. This showed no difference in *FERMT1* between the keratinocyte populations identified in cSCC. *FERMT1* expression did not correlate with pathological differentiation status (Fig. 1C), tumor depth (Fig. 1D), tumor diameter (Fig. 1E) or tumor invasion status (Fig. 1F). Together these data suggest a tumor promoting role for Kindlin-1 in non-KS associated human cSCC.

### Loss of Kindlin-1 leads to increased cSCC invasion and 3D growth

Due to the rarity of KS, there are no models of KS-associated human cSCC to address how Kindlin-1 loss impacts on cSCC behavior. We have previously shown that epithelial specific loss of Kindlin-1 in K14CreER^T2^-Kin1^fl/fl^ mice recapitulates some of the skin defects associated with KS [16, 28] and here we have used cell lines generated from cSCC that developed in K14CreER^T2^-Kin1^fl/fl^ mice (generation of mice described in [28] and cell lines in [29]) following induction of a chemically induced skin carcinogenesis protocol. As described previously, addition of 4-OH tamoxifen allowed genetic deletion of *Fermt1* to generate the cSCC Kin-1^−/−^ cell line (Kin1^−/−^). Wild-type Kindlin-1 (Kin1-WT) was re-expressed in the Kin-1^−/−^ cell line to determine Kindlin-1-dependent effects as described in [29]. As Kindlin-1 is a key regulator of integrin activation, we also expressed a Kindlin-1 mutant into the Kin-1^−/−^ cells (Kin1-AA), which is unable to bind β1 and β3 integrins [30, 31] (Supplementary Fig. 2A, C and generation of cell lines described in [29]).

**Fig. 2:**
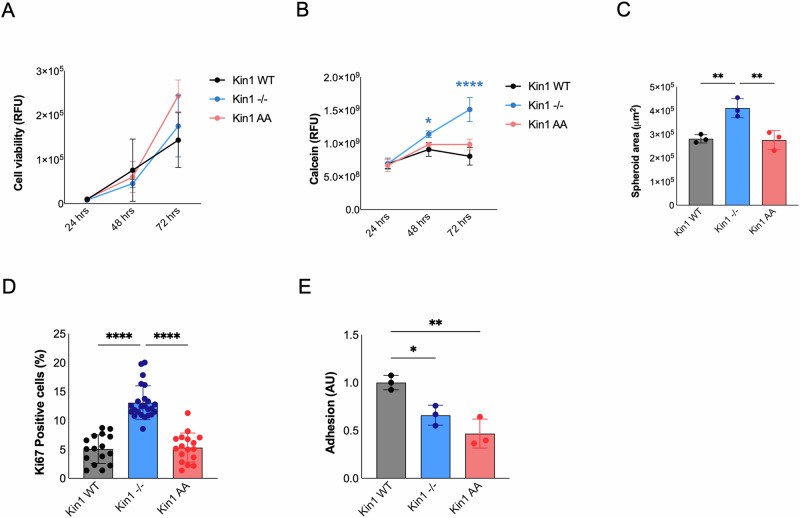
Loss of Kindlin-1 leads to increased cSCC invasion and 3D growth. **A** 2D SCC cell viability over time assessed with AlamarBlue™. **B** Viable cells from cSCC spheroids stained with Calcein-AM at end point. **C** Area of cSCC spheroids after 7 days in culture. **D** Quantification of percentage of Ki67-positive cells in cSCC spheroids. **E** cSCC cell adhesion to fibronectin after 2 h. Data obtained from *n* = 3 (mean ± S. D.). *p*-values were obtained from two-way (**A**, **B**) and one-way ANOVA test (**C**-**E**) followed by Tukey post-hoc test; ****p < 0.0001, **p < 0.01 and *p < 0.05.

Initially we looked at the effect of Kindlin-1 on cSCC proliferation. There was no difference in 2D proliferation of the cells (Fig. 2A) or on the number of apoptotic cells (Supplementary Fig. 2B). However, when grown as 3D spheroids, there was a significant increase in the number of viable cells and spheroid area in the Kin1^−/−^ spheroids (Fig. 2B, C). Immunohistochemical staining of the proliferation marker Ki67 also showed a significant increase in proliferative cells in Kin1^−/−^ spheroids compared to Kin1-WT and Kin1-AA spheroids (Fig. 2D), suggesting that the ability of Kindlin-1 to suppress 3D proliferation is not dependent on its ability to bind and activate β1 integrin. Consistent with impaired integrin activation in the Kin1^−/−^ cells, we saw a reduced adherence to fibronectin, which was dependent on the ability of Kindlin-1 to bind integrins (Fig. 2E). However, flow cytometry with the monoclonal antibody 9EG7, which recognizes an activation-associated epitope on the β1 integrin subunit, showed no difference between the Kin1^−/−^ and Kin1-WT cells, although there was a reduction in the Kin1-AA cells (Supplementary Fig. 2C). The cSCC cells express Kindlin-2 (Supplementary Fig. 2A) and in keratinocytes Kindlin-1 and Kindlin-2 have overlapping functions and can functionally compensate for each other [32]. Kindlin-2 was predominantly localized at focal adhesions in the cSCC cells, which may be sufficient for activation of β1 integrin in the Kin1^−/−^ cells (Supplementary Fig. 3). The downregulation of active β1 integrin in Kin1-AA cells suggests a potential dominant negative effect of this mutant.

### Loss of Kindlin-1 leads to increased tumor growth

**Fig. 3:**
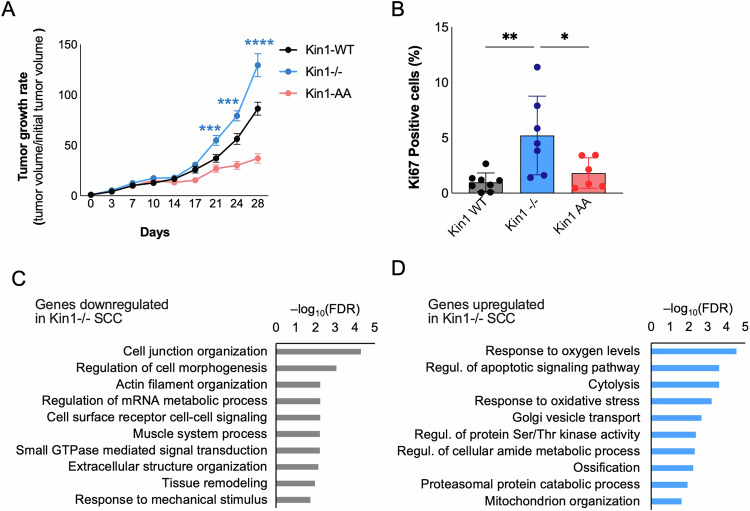
Loss of Kindlin-1 leads to increased tumor growth. **A** In vivo tumor growth rate (tumor volume/initial tumor volume) following subcutaneous injection of cSCC cell lines. p-values were obtained from two-way ANOVA test followed by Tukey post-hoc test; ****p < 0.0001, ***p < 0.001. **B** Quantification of percentage of Ki67-positive cells in cSCC tumors. *p*-values were obtained from one-way ANOVA test followed by Tukey post-hoc test; **p < 0.01 and *p < 0.05. Over-representation of biological processes in the sets of genes significantly downregulated (**C**) and upregulated (**D**) in Kin1^−/−^ tumors.

We then investigated the effects of Kindlin-1 loss on tumor growth in vivo. Kin1^−/−^ tumors grew larger compared to Kin1-WT and Kin1-AA tumors (Fig. 3A). The increased growth of the Kin1^−/−^ tumors was associated with increased expression of the proliferation marker Ki67 (Fig. 3B). The reduced growth of the Kin1-AA tumors compared to the Kin1^−/−^ and Kin1-WT tumors suggests that integrin-dependent signaling plays a role in the in vivo tumor growth.

RNA-Seq analysis of the Kin1-WT and Kin1^−/−^ tumors identified 1202 genes that were differentially expressed (false discovery rate (FDR)-adjusted *p* < 0.05), with 632 genes downregulated and 570 genes upregulated in the Kin1^−/−^ tumors. Genes significantly downregulated in the Kin1^−/−^ tumors were associated with cell adhesion signaling, morphogenesis and actin organization (Fig. 3C), supporting the notion that Kindlin-1 plays an important role in regulating adhesion signaling in these tumors. Genes significantly upregulated in the Kin1^−/−^ tumors were associated with response to oxygen levels and regulation of apoptotic processes (Fig. 3D). Immunohistochemical analysis of cleaved caspase 3 as a marker of apoptosis demonstrated very low levels (< 0.01% cleaved caspase 3-positive cells) in both Kin1-WT and Kin1^−/−^ tumors, with no significant difference between the tumor types, suggesting that the increased growth of the Kin1^−/−^ tumors was not due to decreased levels of apoptosis (Supplementary Fig. 2D). Genes associated with response to oxygen levels included many hypoxia-related genes, such as *Hif1a*, *Hyou1*, *Higd1a*, *Twist1*, *Aqp1*, *Slc2a1*, *Bnip3*, *Ddit4*, *Pdk1*, *Tgfb2* and *Mapk1*, implying an upregulated hypoxia response in the Kin^−/−^ tumors. This is consistent with previous reports that loss of Kindlin-1 promotes a pro-oxidative state in keratinocytes and also cSCC cell lines [29, 33].

**Fig. 4:**
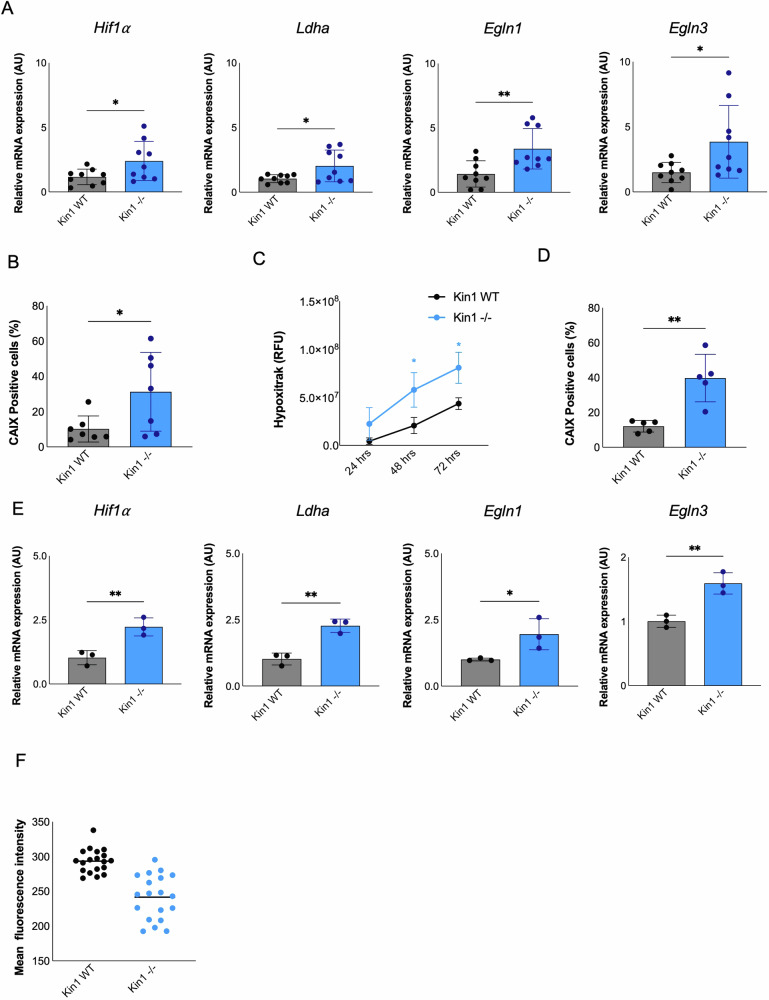
Loss of Kindlin-1 increases gene expression related to hypoxia in cSCC. **A** mRNA expression of hypoxic markers (*Hif1a*, *Ldha*, *Egln1*, *Egln3*) relative to *Actb* in SCC tumors (*n* = 9). **B** Quantification of percentage of CAIX-positive cells in cSCC tumors (*n* = 7). **C** Quantification of HypoxiTRAK™ staining in cSCC spheroids over time (*n* = 3). **D** Quantification of percentage of CAIX-positive cells in cSCC spheroids (*n* = 5). **E** mRNA expression of hypoxic markers (*Hif1a*, *Ldha*, *Egln1*, *Egln3*) relative to *Actb* in cSCC spheroids (*n* = 3). **F** Mitochondria membrane potential in cSCC cells. The values represent the mean±S. D. *p*-values were obtained from two-tailed unpaired *t*-test and from two-way ANOVA test followed by Tukey post-hoc test (C only); **p < 0.01 and *p < 0.05.

To address whether the gene changes associated with hypoxia could be due to the increased tumor size in the Kin1^−/−^ tumors, we collected tumors 7 days post-inoculation when there was no difference in the size between the Kin1^−/−^ and Kin1-WT tumors. There was significantly higher expression of hypoxia-inducible factor 1α (HIF) (encoded by *Hif1a*), and also *Egln1*, *Egln3* and *Ldha*, known HIF target genes, in Kin1^−/−^ tumors compared to Kin1-WT tumors (Fig. 4A and Supplementary Fig. 4A). As we have previously shown that Kin1^−/−^ cells have higher levels of reactive oxygen species (ROS) in response to UV irradiation compared to Kin1-WT cells [29], we asked whether ROS could be contributing to the increased expression of HIF and its target genes. Expression of *Hif1a*, *Egln1*, *Egln2* and *Ldha* was higher in Kin1^−/−^ than Kin1-WT cells grown under hypoxia, and this was attenuated by treatment with the ROS scavenger N-acetyl-L-cysteine (NAC) (Supplementary Fig. 4B).

In addition, immunohistochemical analysis showed increased levels of carbonic anhydrase IX (CAIX), a tumor-associated, cell-surface glycoprotein that is induced by hypoxia, in the Kin1^−/−^ tumors (Fig. 4B). There was a significant increase in the levels of hypoxia in Kin1^−/−^ compared to Kin1-WT spheroids (Fig. 4C) measured by HypoxiTRAK, a cell-permeable dye that fluoresces in hypoxic environments. This was accompanied by an increase in CAIX-positive cells (Fig. 4D) and *Hif1a*, *Egln1*, *Egln3* and *Ldha* expression in spheroids of the same diameter (Fig. 4E and Supplementary Fig. 4C). The *Ldha* gene encodes lactate dehydrogenase A (LDHA), which catalyzes the conversion of pyruvate to lactate and is considered to be a key checkpoint of glycolysis. Together with the increased levels in Kin^−/−^ SCC of CAIX (Fig. 4B, D), which is a marker of adaption to lactic acid production, and of expression of *Pdk1*, a gatekeeper of glycolysis that promotes glycolytic metabolism, this suggests that loss of Kindlin-1 can drive cSCC cells to increased glycolytic ATP production. These data imply a reduced dependence on mitochondrial oxidative phosphorylation for energy supplies in Kin^−/−^ tumors, which is further supported by a significantly reduced mitochondrial membrane potential in Kin1^−/−^ cells (Fig. 4F).

### Loss of Kindlin-1 regulates metalloproteinases

**Fig. 5:**
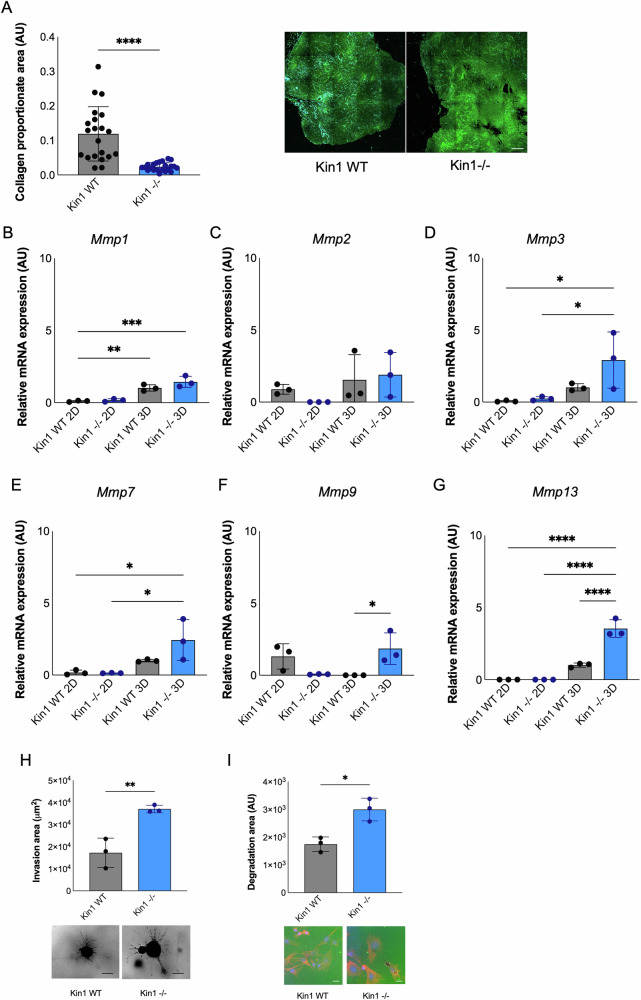
Kindlin-1 loss alters matrix metalloproteinase expression. **A** Quantification of collagen proportionate area (left) with representative SHG images (right) in Kin1-WT and Kin1^−/−^ tumors. Green, GFP; cyan, SHG. Scale bar = 250 µm. *p*-value obtained from two-tailed *t*-test; ****p < 0.0001 from *n* = 21 Kin1-WT and *n* = 23 Kin1^−/−^ tumors. **B**–**G** Expression levels of *Mmp1, Mmp2, Mmp3*, *Mmp7*, *Mmp9* and *Mmp13* in cSCC spheroids relative to *Actb*, normalized to the average expression of 2D Kin1-WT. **H** cSCC spheroid invasion in collagen type I, with representative images after 7 days (bottom panel). Scale bar = 400 µm. **I** Area of cSCC cell degradation of fluorescent gelatin, with representative images (bottom panel) showing intact fluorescent gelatin (green), phalloidin (red) and nuclei (Hoechst). Scale bar = 40 µm. Data obtained from *n* = 3 (mean±S. D.). *p-*values proportionate area (left) with representative were obtained from one-way ANOVA test followed by Tukey post-hoc test (**B**–**G**) and from two-tailed *t*-test (**H**, **I**); ****p < 0.0001, ***p < 0.001, **p < 0.01 and *p < 0.05.

As further analysis of the most highly upregulated genes in the Kin1^−/−^ tumors revealed marked changes in a number of genes that are known to drive tumor invasion and metastatic spread, including collagenases (Table 1), we asked whether Kindlin-1 regulates the collagen content in the cSCC tumors. cSCC cells were inoculated subcutaneously into mice and the resulting tumors collected for analysis of collagen content using second harmonic generation (SHG). There was a significant decrease in the collagen proportionate area in Kin-1^−/−^ tumors compared to Kin1-WT tumors (Fig. 5A). We then profiled the expression of a number of metalloproteinases in the cSCC cells that are known to degrade collagen, including *Mmp3* and *Mmp13*, which were upregulated in Kin^−/−^ tumors. *Mmp3*, *Mmp7*, *Mmp9* and *Mmp13* expression levels were all increased in the Kin1^−/−^ cells compared to Kin1-WT cells when grown as 3D spheroids (Fig. 5B–G and Supplementary Fig. 5A). This was associated with an increased ability of the Kin1^−/−^ cells to invade into collagen gels compared to the Kin1-WT (Fig. 5H). We looked directly at the ability of the cSCC cells to degrade a fluorescently labelled collagen matrix, which showed that collagen degradation was also increased by the Kin1^−/−^ cells compared to the Kin1-WT (Fig. 5I). As MMP13 has been linked to the progression and invasive capacity of cSCC [34–36], we decided to look further at how MMP13 may be contributing to the pro-invasive phenotype of the Kin1^−/−^ cells.

**Table 1:** Upregulated genes in Kin1^−/−^ tumors.

Gene name	Entrez Gene ID	Gene description	Fold upregulation in Kin1^−/−^	TME
*Cstf2*	108062	Cleavage stimulation factor, 3′ pre-RNA subunit 2	65.4	
*Gzmg*	14944	Granzyme G	40.0	+
*Ppp1r10*	52040	Protein phosphatase 1, regulatory subunit 10	32.8	
*Nr4a1*	15370	Nuclear receptor subfamily 4, group A, member 1	26.9	
*Mmp13*	17386	Matrix metallopeptidase 13	22.1	+
*Ggt1*	14598	γ-Glutamyltransferase 1	21.8	
*Adgrf1*	77596	Adhesion G protein-coupled receptor F1	19.6	
*Mmp3*	17392	Matrix metallopeptidase 3	17.8	+
*Ccl9*	20308	Chemokine (C-C motif) ligand 9	16.2	+
*Gzmf*	14943	Granzyme F	15.4	+

Genes upregulated by ≥ 15-fold as determined by RNA-Seq in Kin1^−**/**−^ tumors compared to Kin1-WT tumors are listed. Genes involved in regulating the tumor microenvironment (TME) are labelled with +. Data obtained from three independent experiments; FDR-adjusted *p* < 0.01.

### Kindlin-1-dependent regulation of MMP13 controls SCC invasion

**Fig. 6:**
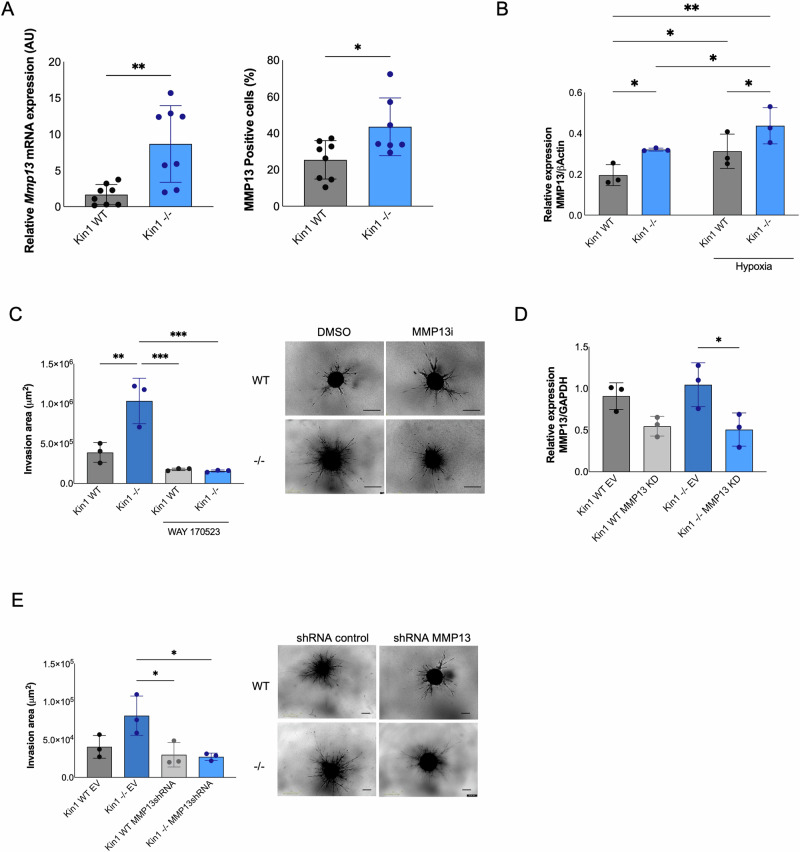
Kindlin-1-dependent regulation of MMP13 controls SCC invasion. **A** *Mmp13* mRNA expression relative to *Actb* (left panel) and percentage of MMP13-positive cells (right panel) in cSCC tumors (*n* = 9). **B** Increased MMP13 expression in cSCC cell lines exposed to hypoxia. Effects of treatment with MMP13 inhibitor WAY 170523 (**C**) or shRNA knockdown of MMP13 (**E**) in SCC spheroid invasion in collagen type I, representative images after 7 days. Scale bar = 400 µm. **D** *Mmp13* mRNA expression in shRNA MMP13 SCC cell lines. Data obtained from *n* = 3 (mean±S. D.). *p*-values were obtained from one-way ANOVA test followed by Tukey post-hoc test; ***p < 0.001, **p < 0.01 and *p < 0.05.

Increased *Mmp13* expression in the Kin1^−/−^ tumors was confirmed, and also verified at the protein level in both tumors and spheroids (Fig. 6A and Supplementary Fig. 5A–D). As previous studies have reported that MMP expression is controlled by an oxidative environment [37], and we had observed increased levels of hypoxia in the Kin1^−/−^ tumors, we then looked at whether hypoxia could induce expression of MMP13. Kin1-WT and Kin1^−/−^ cells incubated under hypoxic conditions had higher levels of MMP13 compared to cells grown under normoxic conditions (Fig. 6B). Furthermore, treatment of the Kin1^−/−^ spheroids with NAC lead to a partial reduction in *Mmp13* suggesting a role for ROS in the control of *Mmp13* by Kindlin-1 (Supplementary Fig. 5E). The *Mmp13* promoter does not contain hypoxia-responsive elements (http://bioit2.irc.ugent.be/contra/v3/#/step/1 [38]) so is unlikely to be a direct HIF target but may be induced indirectly following the expression of other hypoxia-induced pathways in the Kin1^−/−^ cells. To understand further the pathways involved we carried out a proteomic analysis of the Kin1-WT and Kin1^−/−^ cells. This showed that proteins involved in TGFβ signaling were enriched in the Kin^−/−^ cells compared to the Kin1-WT cells (Supplementary Fig. 6A). TGFβ is a key pathway that is active in hypoxic conditions which has been shown to regulate MMP13 expression [39]; treatment with a TGFβ inhibitor attenuated the increased expression of MMP13 in the Kin1^−/−^ cells (Supplementary Fig. 6B).

To determine whether the increased expression of MMP13 in the Kin1^−/−^ cells was contributing to their increased invasion, cSCC spheroids were treated with the MMP13 inhibitor WAY 170523 [40] and invasion into collagen measured. Treatment with the MMP13 inhibitor had no effect on viability (Supplementary Fig. 5F), but prevented the increased invasion of the Kin1^−/−^ spheroids (Fig. 6C). To confirm the effects of the MMP13 inhibitor on invasion, we generated Kin1-WT and Kin1^−/−^ cells in which we knocked down expression of MMP13 using shRNA (Fig. 6D). There was a significant reduction in invasion in the MMP13 shRNA-treated Kin1^−/−^ spheroids compared to the untreated control Kin1^−/−^ spheroids (Fig. 6E). To address whether the effects on invasion were dependent on the ability of Kindlin-1 to bind integrins, invasion of Kin1-AA spheroids was measured. There was no difference in the invasive capacity of the Kin1-AA spheroids compared to the Kin1-WT spheroids, and moreover the expression of MMP13 and levels of hypoxia were unaltered between the Kin1-AA and Kin1-WT spheroids (Supplementary Fig. 7).

## Discussion

Our analysis of human cSCC suggest a role for Kindlin-1 in driving progression of human cSCC where *FERMT1* is increased in cSCC compared to AK and normal skin, and shows an increase in expression with a shift towards a less differentiated and more progenitor-like state. Interestingly *FERMT1* was also identified in an in vivo CRISPR screen in human cSCC xenografts as being pro-tumorigenic [27]. However, KS patients have a higher propensity to develop cSCC. This suggests that the changes seen in the skin of KS patients due to loss of epidermal Kindlin-1 are more important regulators of cSCC development than an increase in Kindlin-1 in pre-cancerous lesions seen in non-KS-associated cSCC. Loss of Kindlin-1 results in increased oxidative stress and the production of proinflammatory cytokines in the skin. Specifically, KS keratinocytes express higher levels of a number of cytokines compared to normal keratinocytes and this can be further enhanced by stresses such as UV irradiation or oxidative stress [18, 19]. In turn factors secreted from KS keratinocytes can lead to activation of fibroblasts [18]. This is consistent with higher levels of fibroblast activation in KS fibroblasts and evidence of fibrosis in the skin of KS patients [17, 18]. Taken together, these changes in the skin of KS patients are associated with a pro-tumorigenic environment.

Using a mouse derived cell line model of Kindlin-1 loss in cSCC, we also showed that loss of Kindlin-1 is associated with increased invasion, a phenotype associated with a more metastatic behavior. In addition, we also see increased tumor growth when Kindlin-1 is depleted. This is consistent with reports of highly aggressive metastatic disease in KS patients. Similar effects were reported in lung cancer, where high Kindlin-1 expression in squamous cell carcinomas is found in well differentiated tumors, and ectopic expression of Kindlin-1 in a lung cancer cell line led to reduced migration and tumor growth in mice [11]. These effects in the lung cancer cell line were associated with a downregulation of epithelial-to-mesenchymal transition (EMT) markers and possible effects on Wnt signaling [11]. Upon Kindlin-1 loss, we saw upregulation of the EMT-associated mesenchymal markers *Twist1, Mmp3 and Mmp13*, as well as downregulation of genes encoding the Wnt signaling components *Wnt9a* and *Wnt10b*, suggesting that a similar mechanism may be involved in cSCC. In esophageal cancer, Kindlin-1 expression is positively correlated with tumor cell differentiation, and higher Kindlin-1 levels were seen in Stage I compared to Stage II and III tumors, suggesting that again Kindlin-1 may also be acting to suppress tumorigenesis in this tumor type [10]. However, in other cancer types such as breast, gastric, colorectal and hepatocellular cancer increased expression of Kindlin-1 is associated with reduced survival, and increased invasive capacity [3–6, 8, 9], which have all been linked to Kindlin-1 dependent induction of an EMT. Interestingly, consistent with our data, immortalized KS keratinocytes had increased levels of EMT markers, and had increased migratory potential compared to normal keratinocytes [20]. Together these studies demonstrate that Kindlin-1 can either induce or inhibit EMT, which is linked to invasive and migratory phenotypes, dependent on the cellular context. Further work is required to understand the role of Kindlin-1 in different cell types.

Although loss of Kindlin-1 did not lead to decreased levels of activated β1 integrin in the SCC cells, the Kin1-AA cells did have lower levels, which most likely reflects the ability of Kindlin-2 in the Kin1^−/−^ cells to compensate for Kindlin-1 loss in mediating integrin activation [3, 32]. Importantly, tumor growth was significantly reduced in the Kin1-AA tumors compared to both the Kin1-WT and Kin1^−/−^ tumors, suggesting that integrin signaling does play a role in vivo. Previously, Kindlin-1 has been reported to have integrin-independent effects on regulation of Wnt signaling in skin (including downregulation of *Wnt9a* expression upon Kindlin-1 loss, as we observe here) [16], and in EGF receptor stability and signaling in keratinocytes [41], although we saw no differences in EGF receptor expression in our SCC model. Kindlin-1 also regulates anti-tumor immunity in an integrin-independent manner through regulation of secreted cytokines that drive integrin-independent immune changes [42].

Loss of Kindlin-1 leads to increased tumor growth, with many of the most upregulated genes in the Kindlin-1-depleted tumors linked to cancer prognosis, including *Cstf2*, *Ppp1r10*, *Nr4a1* and *Ggt1* [43–49]. Interestingly, CSTF2 promotes hepatocellular carcinoma progression and drives glycolysis and lactate production [50]. The increased expression of *Ldha* and *Pdk1* in Kindlin-1-depleted tumors is consistent with increased glycolysis, and LDHA has been reported to facilitate glycolysis and support oral SCC progression [51], while subtypes of acute myeloid leukemia with high PDK1 levels adopt a more glycolytic metabolic state [52].

We and others have previously shown that loss of Kindlin-1 leads to a pro-oxidant state with Kindlin-1-depleted cells having a reduced ratio of oxidized and reduced glutathione (GSSG/GSH) a well-documented marker of oxidative stress and an increased expression of ROS [29, 33]. Here we show that loss of Kindlin-1 leads to the generation of a hypoxic environment within spheroids and tumors, which is known to provide a permissive tumor growth environment. In mouse cSCC tumor models, loss of Hif1α in the epidermis reduced tumor formation in response to UV [53]. However, in human SCC, HIF1α expression is higher in late-stage SCC lesions compared to normal skin and preneoplastic lesions [53, 54], and it is not known how Kin1^−/−^ tumors may utilize a hypoxic environment to promote tumor growth, although we did observe changes associated with glycolytic metabolism. Together this supports a role for loss of Kindlin-1 in promoting a hypoxic pro-tumorigenic environment.

One of the most highly upregulated genes in the Kin1^−/−^ tumors was *Mmp13*, and both pharmacological and genetic inactivation of MMP13 reduced the increased migration and invasion of the Kindlin-1-depleted SCC cells. This is consistent with previous reports which have identified a role for MMP13 in the progression of a number of tumor types, including head and neck and cSCC [35, 55–58]. In addition, serum MMP13 levels have been proposed as a potential diagnostic marker in patients with cSCC [36]. Although nothing is known about MMP13 expression in KS-associated cSCC, its expression in RDEB-associated SCC has been reported, and in a case study of a RDEB patient, MMP13 expression was higher in the cSCC compared to the benign hyperkeratotic lesions [59, 60]. A number of pathways have been reported to regulate *MMP13* expression in SCC, including components of the complement system [35], lncRNAs and miRNAs [34, 58], and growth factors such as TGFβ which we show regulates the Kindlin-dependent expression of MMP13 [61, 62]. Here we have identified a novel role for Kindlin-1 in regulating the hypoxic environment that can contribute to increased expression of MMP13. Targeting MMP13 has been shown to reduce the invasion and growth of cSCC in mice [55, 58] but further work is required to establish whether increased MMP13 levels are associated with cSCC that develop in KS patients. This will require the co-ordinated collection of material from these rare tumors, and will help determine whether this may provide a targeted approach to treating KS-associated cSCC.

## Materials and methods

### RNA expression of FERMT1 against various clinical parameters

Samples from a range of disease progression states in cSCC were obtained for RNA-sequencing [26]. The referred study was approved by the East of Scotland Research Ethics Service (REC reference 08/S1401/69), The Ethics and Scientific Committee of A. Sygros Hospital (Ref 2353/3-11-2016) and The University of California, San Francisco Institutional Review Board and conducted according to the Declaration of Helsinki Principles. All patients participating in the study provided written, informed consent. Punch biopsies of samples were snap frozen in liquid nitrogen. Gene counts from this were normalized by the DESeq2 R package using the variance stabilizing transformation (vst) function and R package ggplot2 was used to generate bar plots, boxplots and scatterplots of *FERMT1* expression across various clinical parameters [63, 64, 65]. In the boxplots, boxes encompass the interquartile range (IQR) around the median and the whiskers display values within 1.5 * IQR from the upper and lower quartiles. Datapoints past the whiskers are considered outliers. Global significance values in the boxplots were ascertained using the Kruskal-Wallis test and the significance of pairwise comparisons were calculated using two-sided Wilcoxon rank-sum tests from R package ggpubr [66]. Linear models in the scatterplots were generated using the lm function from the Rstudio base package, stats.

### Cell lines and drugs

Kindlin-1 wild type (Kin1-WT), Kindlin-1 knockout (Kin1^−/−^) and the non-integrin binding Kindlin-1 mutant (Q611A/W612A) (Kin1-AA mutant) cell lines were generated and grown as described previously [28, 29]. Cells were routinely tested for mycoplasma every month with a Mycoalert® Mycoplasma detection kit (Lonza, Little Chesterford, United Kingdom). Knockdown of MMP13 was achieved with the use of shRNA constructs in vector pGFP-C-shLenti (TR30023) from OriGene Technologies (Rockville, MD, USA) and is described in the Supplementary material. MMP13 inhibitor WAY1700523 was purchased from Tocris Bioscience (Bristol, United Kingdom). 10 mM stock solutions in dimethyl sulfoxide (DMSO) were stored at 4 °C and diluted to a concentration of 10 µM for experiments.

### Spheroid formation

2000 cells were plated in U-bottom ultralow-attachment plates, centrifuged at 1000 × *g* for 10 min with low acceleration and deceleration and incubated for 48 h to generate spheroids. For RNA and protein extraction, spheroids were grown in MicroTissues® 3D Petri Dish® micro-mold spheroids (Sigma-Aldrich) in 24-well plates for 48 h, based on the manufacturer’s instructions. For immunohistochemistry, spheroids grown in molds were fixed with 4% w/v PFA for 24 h, covered in 2% w/v Invitrogen™ UltraPure™ agarose (Thermo Fisher Scientific, Waltham, MA, USA) and embedded in paraffin.

### Spheroid staining

To assess hypoxia, SCC cells were plated in the presence of 100 nM Hypoxitrak™ (Biostatus, Leicestershire, United Kingdom) and Hoechst 33342 (1.0 µM; Thermo Fisher Scientific). For endpoint studies, Calcein-AM (2.5 µM; Thermo Fisher Scientific) was added and spheroids were incubated for 1 h. All imaging was performed on an ImageXpress Micro XLS High-content analysis system (Molecular Devices, San Jose, CA, USA).

### 3D invasion

To assess invasiveness, medium was removed from formed spheroids and rat tail collagen type-I (Corning) was added (0.5 mg/ml) with pre-chilled tips. Plates were centrifuged at 300 × *g* for 5 min at 4 °C to ensure central location of spheroids. After 1 h at 37 °C, complete medium was added to generate a chemoattractive gradient. Cell invasion was monitored on an IncuCyte® S3 system over 7 days, and spheroid invasion analysis was performed using the IncuCyte® S3 Spheroid Software module (Essen Bioscience, Ann Arbor, MI, USA).

### Substrate degradation

To quantify substrate degradation, 96-well plates were coated with 35 μg/ml fluorescent conjugate of gelatin from pig skin (Thermo Fisher Scientific) for 1 h at 37 °C. Next, wells were washed with PBS and 2000 cells were plated and incubated in normal culture conditions. After 72 h, cells were fixed with 4% w/v PFA for 20 min and permeabilized with 0.1% w/v Triton X-100 for 5 min. Cells were stained with AlexaFluor 568 phalloidin (1:2000; Thermo Fisher Scientific) for 30 min and Hoechst 33342 (1:1 000; Thermo Fisher Scientific) for 15 min before imaging on an ImageXpress Micro XLS High-content analysis system. Area degradation was quantified using FIJI software.

### Cell adhesion

Adhesion assays were performed in 96-well plates coated with 20 μg/ml human fibronectin (Corning) overnight at 4 °C. Plates were washed with PBS, blocked for 1 h with 1% w/v BSA in DMEM and washed with PBS before use. Cells were seeded (3 × 10^4^ per well) and allowed to adhere for 1 h at 37 °C. Plates were rinsed with PBS and cells were fixed with 4% w/v PFA for 20 min at room temperature. Plates were washed with water and stained with 0.5% w/v crystal violet (Sigma-Aldrich) for 10 min. Plates were washed with water twice and adherence was measured by absorbance (490 nm) on a microplate reader (Tecan, Männedorf, Switzerland).

### RT-qPCR

Tumor tissue specimens were snap-frozen and stored at −80 °C prior to homogenization by sterile scalpels and, for tissue culture, cell pellets were obtained. RNA extraction was performed with a RNeasy® Mini Kit (QIAGEN, Venlo, Netherlands) based on the manufacturer’s instructions, including treatment with RNase-Free DNAse kit (QIAGEN). Samples were resuspended in RNase-free water (QIAGEN) and quantified with a Nanodrop 2000 (Thermo Fisher Scientific) with a 260/280 ratio between 2.0–2.2. 0.5 μg of RNA was retrotranscribed with SuperScript™ II First-Strand Synthesis System (Thermo Fisher Scientific) based on the manufacturer’s instructions with Oligo-dT provided on the kit. qPCR was performed based on SYBR™ Select Master Mix protocol (Thermo Fisher Scientific) using the StepOne Real-Time PCR System (Applied Biosystems, Waltham, MA, USA) as follows: 95 °C for 10 min for denaturation, followed by 40 cycles of 95 °C for 15 s for amplification, annealing 53 °C for 30 s and extension at 72 °C for 30 s, melt curve stage at 95 °C for 15 s, 60 °C for 1 min and 95 °C for 15 s. RNAse-free water and no retrotranscribed control RNA samples were used as negative controls. Quantification cycle values were normalized to *Actb* and *Gapdh* expression in separate experiments and relative quantification of expression levels was performed using the 2^−ΔΔCt^ method. For *Mmp13* expression, quantification was normalized to the mean value of two reference genes (*Actb* and *Rps18*) with the use of the 2^−ΔΔCt^ method. Primer sequences obtained from IDT (Coralville, IA, USA) are listed in Supplementary Table 2.

### Mice

All experiments were carried out in compliance with UK Home Office regulations and were approved by the University of Edinburgh Animal Welfare and Ethical Review Body (PL05-21). cSCC cells (0.25 × 10^6^) were injected subcutaneously into both flanks of 8–12 week-old female CD-1 Nude mice (Charles River Laboratories, Wilmington, MA, USA) (*n* = 9), and tumor growth was measured twice weekly using calipers. Tumor volume was calculated using the formula *V* = (*W*^2^ × *L*)/2, where *V* is tumor volume, *W* is tumor width and *L* is tumor length. Tumor growth rates were calculated from the tumor volume measurements at each time point expressed as a ratio of the initial tumor volume. Results from two separate experiments, each with 4 mice per group carrying bilateral tumors. Tumors were collected at sacrifice and processed for RNA-Seq, RT-qPCR or immunohistochemical analysis. For 7-day experiments, mice were injected with 1 × 10^6^ cSCC cells.

### Statistical analysis

All statistical analyses and graphs were performed and generated using Prism version 9.3.0 (GraphPad, San Diego, CA, USA), and all data are presented as the mean ± SD. For most, in vitro *n* = 3 and due to this, individual data points were plotted. Statistical analysis for two groups of data from a single experiment were determined by unpaired *t*-test, differences between more than two groups with one or two independent variables were assessed by one-way or two-way ANOVA, respectively, followed by Tukey post-hoc test with multiple comparisons between groups. p-values to determine statistical significance are indicated in the text.

### Supplementary information

Supplementary Material

## Data Availability

Datasets related to this article can be found at https://www.ncbi.nlm.nih.gov/geo/query/acc.cgi?acc=GSE236883, hosted at Gene Expression Omnibus (GSE236883).
