# Supplementary material for: Involvement of Kindlin-1 in cutaneous squamous cell carcinoma
Source: Oncogenesis. 2024 Jul 9;13(1):24. doi: 10.1038/s41389-024-00526-1 (PMC11233684; doi:10.1038/s41389-024-00526-1)
Supplement: Supplementary file 1 — Supplementary Material [file 41389_2024_526_MOESM1_ESM.docx]

**Supplementary Materials and Methods**

**MMP13 knockdown cell lines**

A non-targeting empty vector (EV) was used as a control (Supplementary Table 1). Briefly, LentiX 293T cells were transfected with jetOPTIMUS® DNA transfection reagent (Illkirch, France), based on the manufacturer’s instructions, with lentiviral vectors psPAX2 packaging plasmid (0.6 μg/μl), pMD2.G envelope plasmid (0.8 μg/μl) obtained from Addgene (Watertown, MA, USA), and shRNA constructs (0.25 μg/μl). After 24 hours, the medium was refreshed, and the following day, medium containing lentiviral particles was centrifuged and stored at −80°C. cSCC cells (5x10^5^cells) were incubated with lentiviral particles (1:3) and polybrene (4 μg/ml) from Sigma-Aldrich (Burlington, MA, USA). After 24 hours, virus-containing transduction medium was discarded and replaced with fresh medium for an additional 48 hours. Medium was refreshed, and all MMP13-knockdown cell lines were selected by fluorescence-activated cell sorting (BD FACS ARIA II; BD Biosciences, Franklin Lakes, NJ, USA) based on positive GFP expression and, additionally, treatment with puromycin (8 μg/ml) obtained from Gibson Bioscience (Lexington, KY, USA). RT-qPCR analysis was conducted to select the most efficient knockdown of MMP13 out of four constructs.

**2D cell viability**

SCC cells (2 000 per well) were plated in 96-well plates in standard growth medium. After specified timepoints, 10% v/v AlamarBlue® reagent (Thermo Fisher Scientific) was added and incubated for 2 hours. Fluorescence (excitation 570 nm, emission 600 nm) was measured on a microplate reader (Tecan, Männedorf, Switzerland). A blank absorbance value, from wells without cells and treated with AlamarBlue®, was subtracted from all values to eliminate background fluorescence.

**Immunohistochemistry**

Formaldehyde-fixed tumor samples were embedded in paraffin, sectioned onto slides at a thickness of 5 μm and rehydrated with xylene and decreasing gradients of ethanol. For antigen retrieval, samples were treated with 0.1 M citrate buffer (pH 6.0) at 100°C and blocked with peroxidase blocking solution (Agilent, Santa Clara, CA, USA) for 5 minutes and with serum-free protein solution (Agilent) for 10 minutes at room temperature. Primary antibodies (1:800 Ki67 for tissue and 1:100 for spheroids and 1:100 caspase 3 from Cell Signaling Technology, Danvers, MA; 1:800 carbonic anhydrase-9 from Abcam, Cambridge, United Kingdom; 1:1 000 MMP13 from Thermo Scientific) were incubated overnight at 4°C. Sections were washed 2 times with 0.05% v/v TBS-T, incubated with EnVision System HRP labelled polymer anti-rabbit (Agilent) for 30 minutes, washed 2 times with 0.05% v/v TBS-T and incubated with DAB+ (Agilent) for 5 minutes. Next, samples were counterstained with Mayer’s hematoxylin (Sigma-Aldrich) for 2 minutes and exposed to increasing gradients of ethanol and xylene before mounting with DPX (Sigma-Aldrich). Immunostained tissue slides were digitized using NanoZoomer slide scanner (Hamamatsu, Shizuoka, Japan) and positive cell percentage was obtained using QuPath software analysis (version 0.4.3.).

**Western blotting**

For hypoxia studies, cells were incubated for 24 hours in a humidified incubator at 37°C in 3% v/v oxygen prior to lysis. Protein extraction was performed with RIPA buffer (50 mM Tris-HCl, pH 7.4, 150 mM NaCl, 1% w/v Triton X-100, 0.5% w/v sodium deoxycholate, 0.1% w/v SDS; all from Sigma-Aldrich, Burlington, MA, USA) supplemented with protease (2% v/v) and proteinase (0.4% v/v) inhibitors (Sigma-Aldrich), and quantified by Pierce™ bicinchoninic acid protein assay, according to the manufacturer’s instructions (Thermo Fisher Scientific, Waltham, MA, USA). Proteins (20 μg) were separated according to size with Mini-Protean® TGX Precast gels (Bio-Rad, Hercules, CA, USA), transferred onto nitrocellulose membrane and blocked for 1 hour in TBS containing 0.05% w/v Tween-20 (TBS-T) and 5% w/v BSA (EMD Millipore, Burlington, MA, USA). Membranes were incubated overnight at 4°C with primary antibodies (1:2 000 for Kindlin-1 and 1:4 000 for COX2 from Abcam, Cambridge, United Kingdom; 1:1 000 for MMP13, 1:1 000 for β-Actin, 1:2 000 for Kindlin-2 and 1:1 000 for GAPDH from Cell Signaling Technology, Danvers, MA, USA), washed with TBS-T and incubated with 1:3 000 secondary anti-rabbit IgG HRP-linked antibody (Cell Signaling Technology) for 1 hour. After three washes, membranes were incubated with Clarity Western ECL blotting substrate (Bio-Rad) and visualized using a ChemiDoc Imaging System (Bio-Rad). All antibodies were diluted in 5% w/v BSA in TBS-T.

**Flow cytometry**

Cells were washed with ice-cold PBS, trypsinized and resuspended in 1% w/v BSA in PBS. To maintain integrin expression on the cell surface, cells were maintained in 4°C after harvesting. SCC cells were incubated with 1:100 CD29-PE (Thermo Fisher Scientific) to assess total expression of integrin β1 or 1:100 9EG7 (BD Pharmingen, San Diego, CA, USA) for activated integrin β1 expression. Fluorescence minus control and cells without antibody were used as controls for gating. Apoptosis was assessed using a dead cell apoptosis kit with annexin-V FITC and propidium iodide, based on the manufacturer’s instructions (Thermo Fisher Scientific). All flow cytometry data were collected using a BD LSR-Fortessa X20 and were analyzed using FlowJo™ software (BD Biosciences, Franklin Lakes, NJ, USA).

**Immunofluorescence**

Cells were fixed and permeabilized on ice for 10 minutes in cold fixation solution (3.7% w/v formaldehyde (Thermo Fisher Scientific) and 100 mM PIPES, 1 mM MgCl_2_, 10 mM EGTA and 0.2% w/v Triton X-100 (Sigma-Aldrich)). Cells were then washed 3 times on ice with 0.1% w/v Triton X-100 in TBS and blocked for 1 hour on ice with 2% w/v BSA (EMD Millipore) in TBS-T before overnight incubation at 4°C with primary antibodies (1:100 Kindlin-1 from Abcam; 1:200 Kindlin-2 from Sigma-Aldrich; 1:200 FAK from Cell Signaling Technology) in 2% w/v BSA in TBS-T. Following 3 washes with TBS-T on ice, cells were incubated for 45 minutes on ice in the dark with secondary fluorescent antibodies (488 or 594 Alexa Fluor from Invitrogen, Waltham, MA, USA). Finally, coverslips were washed 3 times on ice in the dark with TBS-T, before being mounted with DAPI-containing Vectashield mounting medium (Vector Laboratories, Newark, California). Visualization of immunofluorescence staining was performed using an Olympus FV1000 confocal microscope (Olympus, Tokyo, Japan) with a 60× 1.35 NA UPLANSAPO oil immersion objective, and Olympus FluoView FV1000 software (Olympus).

**Mitochondrial membrane potential**

cSCC cells (3x10^4^) were plated on sterile 19 mm round glass coverslips. When reaching optimal density (24-48 hours post-seeding), cSCC cells were incubated in media supplemented with 100nM MitoTrackerTM Red CMXRos (Life Technlogies, Carlsbad, CA, USA) for 45 minutes at 37°C and fixed in fixation buffer for 10 minutes at 37ºC. Cells were incubated in glycine for 10 minutes to quench residual formaldehyde, washed twice in wash buffer and blocked with 2% w/v BSA (EMD Millipore, Burlington, MA, USA) in TBS-T as a blocking buffer for 1 hour at RT. Cells were incubated overnight at 4ºC with anti-COX IV (1:200, Cell Signaling Technology), washed 3 times with 0.1% w/v Triton X-100 in TBS and incubated with Alexa Fluor secondary antibody 488 (1:300, Thermo Fisher Scientific) and Phalloidin-Atto 647N (1:300, Sigma-Aldrich) for 1 hour at RT. Next, cells were washed 3 times with 0.1% w/v Triton X-100 in TBS followed by one wash with distilled water. Finally, coverslips were mounted with DAPI-containing ProLong™ Glass Antifade Mountant (Thermo Fisher Scientific). To measure membrane potential, 19 images from different fields of view were captured with FV3000 confocal microscope (Olympus) and processed with FIJI software. COX IV staining was used to select mitochondria areas. Membrane potential was quantified from mean florescence intensity of MitoTracker CMXRos from COX IV positive areas. cSCC cells treated with 20 μM FCCP were used as a control for membrane potential.

**Collagen analysis**

Second harmonic generation (SHG) signals were acquired from cSCC tumors as described previously [63]. Images were recorded using FV10-ASW software (Olympus, Tokyo, Japan) and the Multiarea function to record a 5×5 overlapped grid of 512×512 pixel images with a 10 μs per pixel dwell time and line integration of 2. Images were stitched together in the FV10-ASW software before being processed in ImageJ (National Institutes of Health, Bethesda, MD, USA). Images were inspected and clear regions of skin removed manually. Tumor regions were defined by applying a *MinError* binary threshold to GFP-stained images, then removing small (<250-pixel) regions and creating a tumor region of interest (RoI). Collagen regions were defined by applying either a default binary or *Renyi Entropy* binary threshold to SHG images. The *Image Calculator* function was applied to the binary images to determine regions of collagen within the tumor region and convert them into an RoI. Proportion of collagen was calculated by dividing the area of the tumor collagen RoI by the area of the tumor RoI.

**Proteomics**

Cell pellets from Kin1 WT and Kin1^-/-^ cSCC cells were lysed in 50 µl whole proteome lysis buffer (6M GuHCl, 100mM TrisHCl 8.5, 1mg/ml Chloracetamide, 1.5 mg/ml TCEP). Lysate was sonicated with a probe sonicator (Soniprep 150) until no longer viscous, and boiled at 95°C for 5 min, then centrifuged at 14 000 rpm for 5 min. Supernatant was then transferred to a fresh tube and pre-digested with Lys-C (Fujifilm WakoPure Chemical Corporation, Japan) added at the concentration of 1µg/sample, then samples incubated at 37°C for 3 hours. Samples were diluted 1:5 by addition of 200 µl Mass Spec grade water, and 1µg trypsin (Promega, Madison, WI, USA) was added to each sample. Samples were incubated at 37°C with shaking overnight. Samples were acidified with 1% trifluoroacetic acid (TFA) and centrifuged at 13 000 rpm, for 10 minutes at room temperature. Sample was applied to a double layer Empore C18 Extraction Disk (3M GmbH, Germany) prepared with methanol. Membrane was washed twice with 0.1% TFA and protein was eluted with elution buffer (50% acetonitrile (ACN), 0.05% TFA), dried using a CentriVap Concentrator (Labconco, Kansas City, MO, USA) and resuspended in 15 µl 0.1% TFA. Protein concentration was determined by absorption at 280nm on a Nanodrop 1000, then 2 µg of de-salted peptides were loaded onto 25cm Aurora Columns (IonOptiks, Australia) using a RSLC-nano uHPLC systems connected to a Q Exactive mass spectrometer (both Thermo Scientific, United Kingdom). Peptides were separated by a 70 min linear gradient from 5% to 30% acetonitrile, 0.5% acetic acid. The mass spectrometer was operated in DIA mode, acquiring a MS 350-1 650 Da at 120k resolution followed by MS/MS on 45 windows with 0.5 Da overlap (200-2 000 Da) at 30 k with a NCE setting of 28. Raw files were analyzed and quantified using DIA-NN against the Uniprot Mus Musculus database with the default settings.

Data was analyzed on Perseus software (version v2.0.9.0) by two-sample T test and differentially expressed genes (p values < 0.05) were used for preranked GSEA analysis with reference gene sets database from Molecular SignaturesDatabase (MSigDB) of mh.all.v2023.2.Mm.symbols.gmt.

**3D treatment with inhibitors**

Kin1^-/-^ cells were grown as spheroids as specified in the general methods and treated with 1 μM ERK inhibitor (U0126; InVivoGen, San Diego, CA, USA), p38 inhibitor (SB 203580; Sigma-Aldrich), or TGFβ receptor inhibitors SB-431542 (MedChemExpress, Monmouth Junction, NJ, USA) or Vactosertib (MedChemExpress) for 48 hours. Cell lysis was performed as specified in the western blot method from this section.

For assessing the effects of ROS, SCC cells were grown as spheroids as specified in the general methods. After 48 hours, spheroids were treated with 10 μM N-acetylcysteine (Sigma-Aldrich) or DMSO and incubated in a humidified incubator at 37°C in 3% v/v oxygen for 24 hours before extracting RNA as specified in the general methods.

1. Trapnell, C., et al., *Differential analysis of gene regulation at transcript resolution with RNA-seq.* Nat Biotechnol, 2013. **31**(1): p. 46-53.

**Supplementary tables**

**Supplementary Table 1. *Mmp13* shRNA constructs and target sequence**

| **Name** | **Construct** | **Target sequence** |
| --- | --- | --- |
| *Mmp13* | TL511095C | TCTGGAGTAATCGCATTGTGAGAGTCATG |
| Scrambled negative control | TR30021 |  |

**Supplementary Table 2. Primers used for RT-qPCR.**

| **Gene** | **Forward primer (5′­–3′)** | **Reverse primer (5′–3′)** |
| --- | --- | --- |
| *β Actin* | GGCTGTATTCCCCTCCATCG | CCAGTTGGTAACAATGCCATGT |
| *Gapdh* | CAGTGCCAGCCTCGTCCCGTAGA | CTGCAAATGGCAGCCCTGGTGAC |
| *RPS18* | CAGCCAGGTTCTGGCCAACGG | ATACACCCACAGTTCGGCCCCTG |
| *Hif1a* | CGCTATCCACATCAAAGCAA | GCACTAGACAAAGTTCACCTGAGA |
| *Ldha* | TGTGGCAGACTTGGCTGAGA | CTGAGGAAGACATCCTCATTGATTC |
| *Egln1* | GACCGGCGTAACCCTCATG | TTGCTGACTGAATTGGGCTTG |
| *Egln3* | TCAACTTCCTCCTGTCCCTCATC | GCGAACATAACCTGTCCCATTTC |
| *Mmp1* | AGGAAGGCGATATTGTGCTCTCC | TGGCTGGAAAGTGTGAGCAAGC |
| *Mmp2* | AAGGATGGACTCCTGGCACATGCCTTT | ACCTGTGGGCTTGTCACGTGGTGT |
| *Mmp3* | CCAAGTCTAACTCTCTGGAACCTG | AGAGATTTGCGCCAAAAGTG |
| *Mmp7* | ACTTCAGACTTACCTCGGATCG | TCCCCCAACTAACCCTCTTGA |
| *Mmp9* | AAGGACGGCCTTCTGGCACACGCCTTT | GTGGTATAGTGGGACACATAGTGG |
| *Mmp13* | GCCAGAACTTCCCAACCAT | TCAGAGCCCAGAATTTTCTCC |

**Supplementary Table 3. List of antibodies.**

| **Name** | **Catalog number** | **Vendor** | **Dilution** | **Purpose** |
| --- | --- | --- | --- | --- |
| Ki67 | 12202S | Cell Signaling Technology | 1:800 tissue/1:100 spheroids | Immunohistochemistry |
| Caspase 3 | 9664S | Cell Signaling Technology | 1:100 | Immunohistochemistry |
| Carbonic anhydrase 9 | ab243660 | Abcam | 1:800 | Immunohistochemistry |
| MMP13 | ab51072 | Abcam | 1:1 000 | Immunohistochemistry/Western blot |
| Kindlin-1 | ab68041 | Abcam | 1:2 000/1:100 | Western blot/Immunofluorescence |
| Kindlin-2 | K3269 | Sigma-Aldrich | 1:2 000/1:200 | Western blot/ Immunofluorescence |
| COX2 | ab15191 | Abcam | 1:4 000 | Western blot |
| GAPDH | 5174S | Cell Signaling Technology | 1:1 000 | Western blot |
| β-Actin | 3700S | Cell Signaling Technology | 1:1 000 | Western blot |
| CD29-PE | 12-0291-82 | Thermo Scientific | 1:100 | Flow cytometry |
| 9EG7 | 553715 | BD Biosciences | 1:100 | Flow cytometry |
| FAK | 3285S | Cell Signaling Technology | 1:200 | Immunofluorescence |

**Supplementary figures**

**
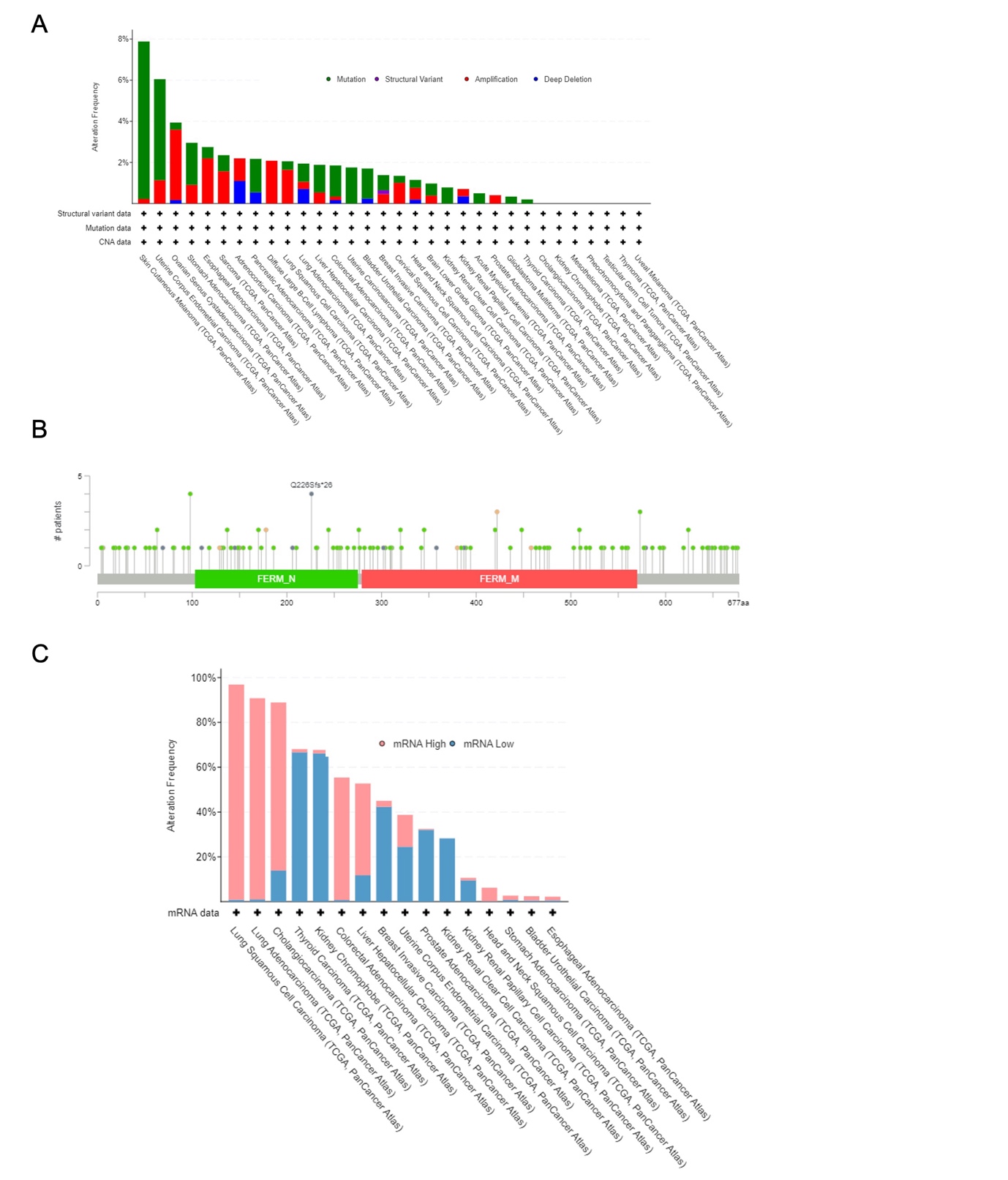
**

**Supplementary Figure 1. Pan-cancer analysis of *FERMT1* alterations.** cBioPortal analysis of the TCGA pan-cancer cohort revealed that (A) *FERMT1* is infrequently mutationally altered in most tumor types. (B) Localization of missense mutations in the coding region of *FERMT1.* (C) Overexpression of *FERMT1* mRNA is observed in lung, colorectal cholangiocarcinoma and hepatocellular carcinomas and downregulation of expression is observed in thyroid, kidney, breast, endometrial and prostate tumors.

**
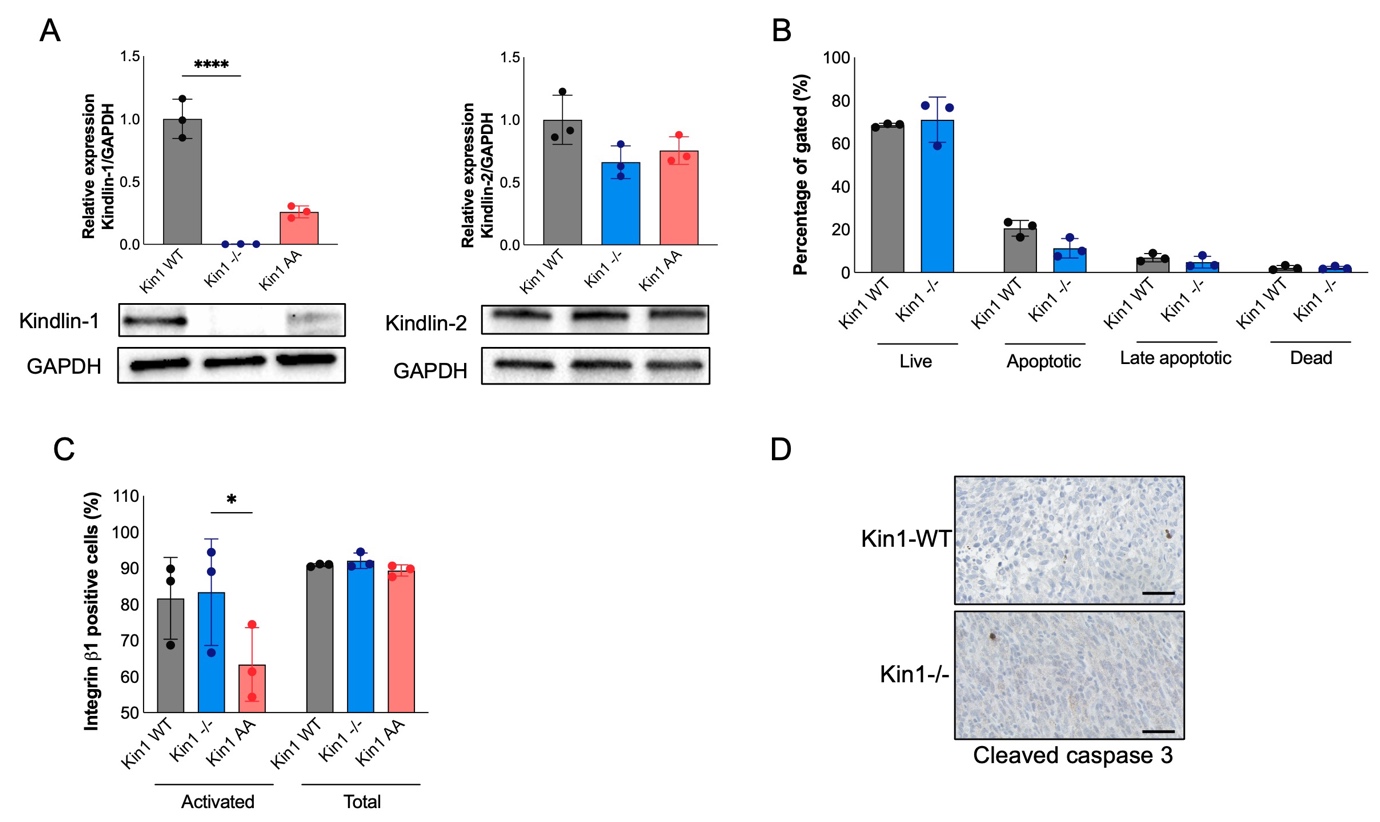
**

**Supplementary Figure 2. Characterization of cSCC cell lines.** (A) Expression of Kindlin-1 and Kindlin-2 relative to GAPDH in cSCC cell lines, assessed by Western blot. (B) Percentage of apoptosis in cSCC cells grown in 2D, assessed by flow cytometry. (C) Flow cytometry quantification of cell-surface expression of active and total integrin 1β in cSCC cells. Data obtained from n=3 (mean ± S. D.). p-values were obtained from one-way ANOVA test followed by Tukey post-hoc test; ****p < 0.0001 and *p < 0.05. (D) Representative IHC images of cleaved caspase-3 staining in cSCC tumors. Scale bar = 50 µm.

**
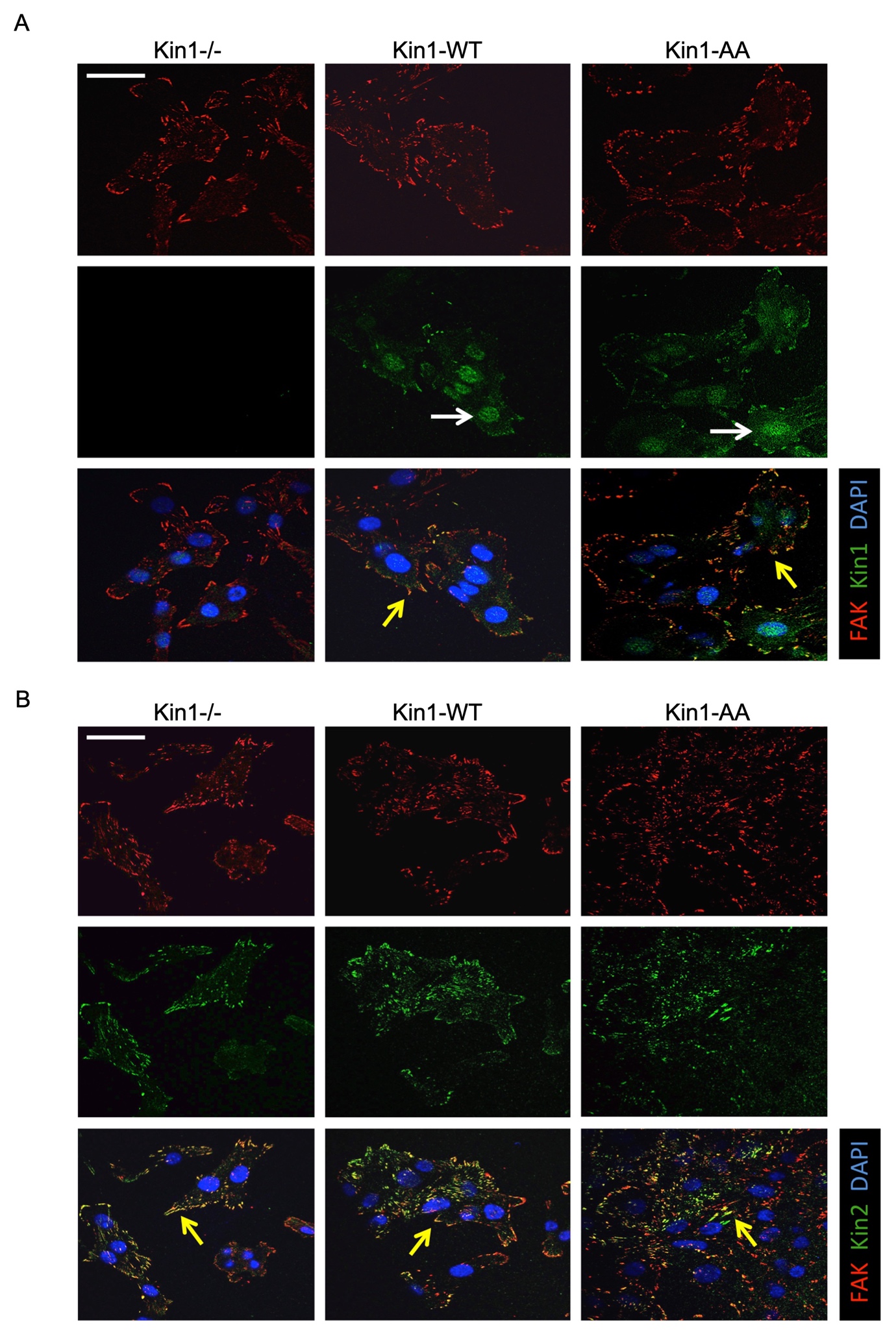
**

**Supplementary Figure 3. Subcellular localisation of Kindlin-1 and Kindlin-2 in cSCC cells.** Representative immunofluorescence images of Kindlin-1 (A) and Kindlin-2 (B) subcellular localisation (green), FAK (red) and nuclei (DAPI) in cSCC cell lines. White arrows indicate nuclear localisation of Kindlin-1. Yellow arrows indicate colocalization of Kindlin-1 or Kindlin-2 with FAK at focal adhesions. Scale bar = 50 µm.

**
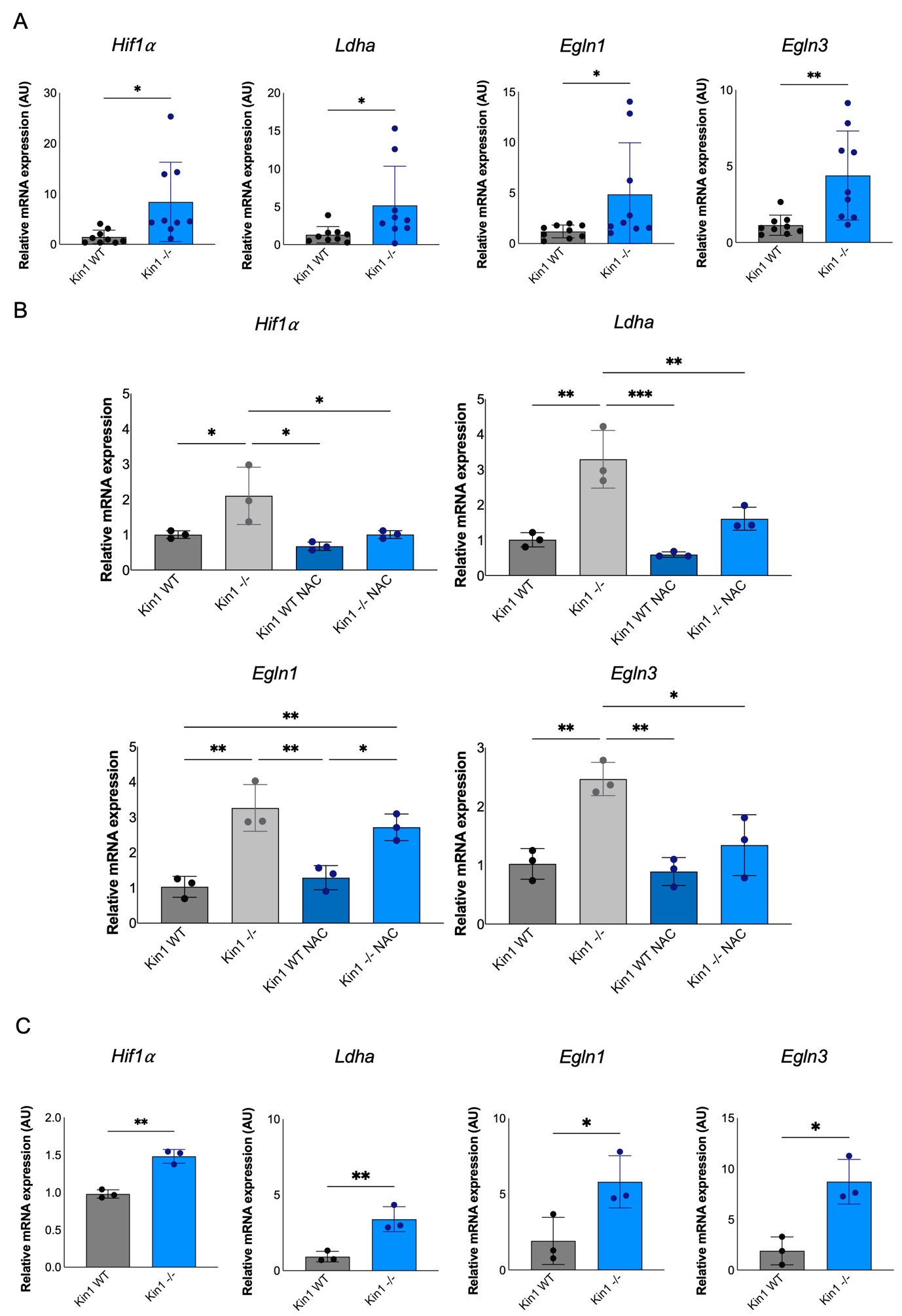
**

**Supplementary Figure 4.** (A) mRNA expression of hypoxic markers (*Hif1a*, *Ldha*, *Egln1*, *Egln3*) relative to *Gapdh* in SCC tumors (n=9). (B) mRNA expression of hypoxic markers (*Hif1a*, *Ldha, Egln1*, *Egln3*) relative to *Actb* in cSCC spheroids treated with DMSO or the ROS scavanger N-acetylcysteine for 24 hours (n=3). (C) mRNA expression of hypoxic markers (*Hif1a*, *Ldha*, *Egln1*, *Egln3*) relative to *Gapdh* in cSCC spheroids (n=3). The values represent the mean ± S. D. p-values were obtained from two-tailed unpaired *t*-test (for A and C) and from one-way ANOVA test followed by Tukey post-hoc test (for B); ***p<0.001, **p < 0.01 and *p < 0.05.

​

**
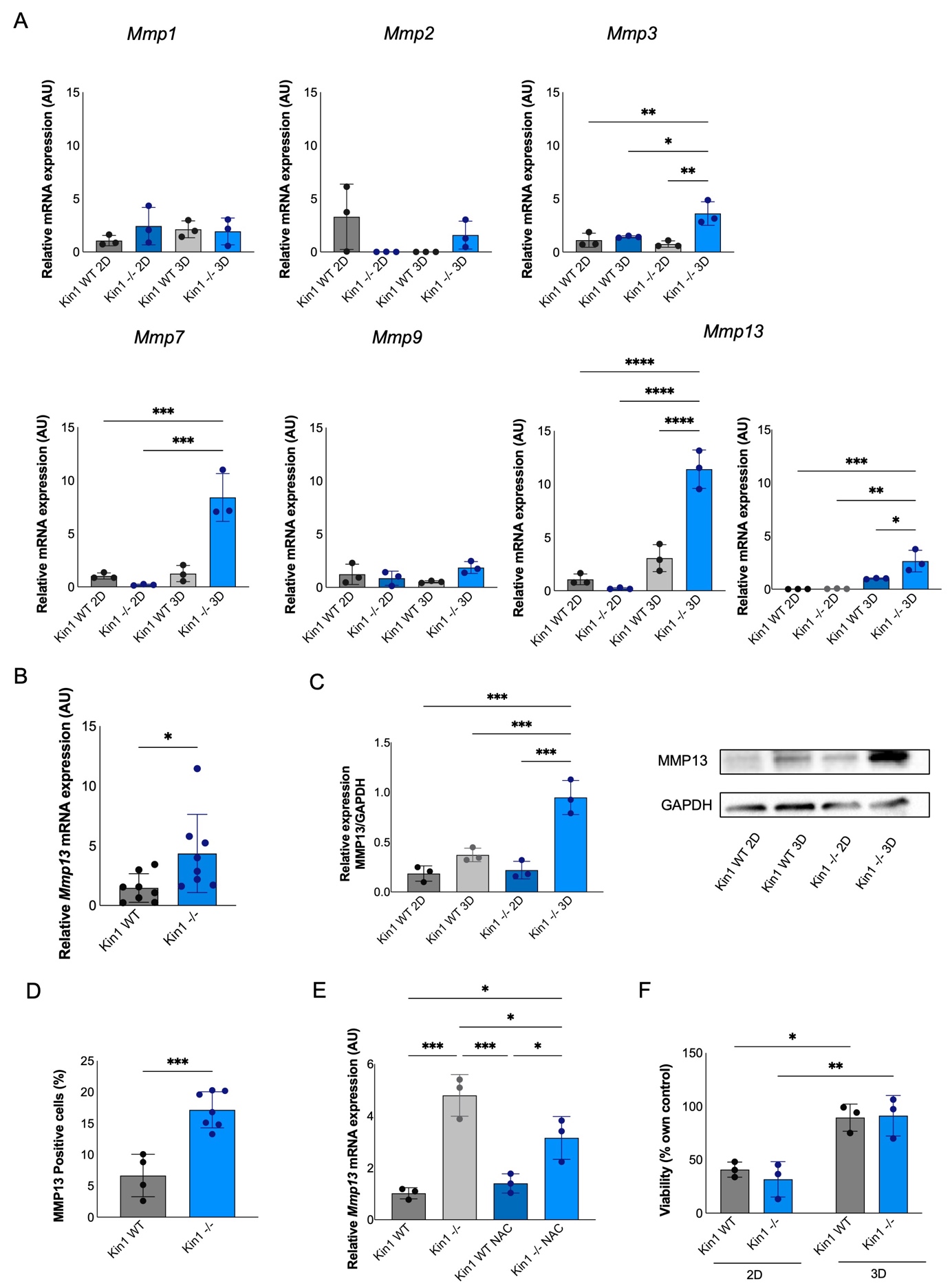
**

**Supplementary Figure 5. MMP13 expression in SCC cells and tumors.** (A) Expression levels of *Mmp1, Mmp2, Mmp3*, *Mmp7*, *Mmp9* relative to *Gapdh* and *Mmp13* relative to *Gapdh* (left hand graph) and both *Actb* and *Rsp18* (right hand graph) in cSCC spheroids, normalized to the average expression of 2D Kin1-WT. Data obtained from n=3 (mean ± SD). (B) *Mmp13* mRNA expression relative to *Actb* and *Rsp18* in cSCC tumors, n=9. (C) Expression of MMP13 relative to GAPDH in SCC cell lines grown in 2D and 3D, assessed by Western blot. (D) Percentage of MMP13-positive cells in SCC spheroids, assessed with immunohistochemistry. (E) Expression of *Mmp13* relative to *Actb* and *Rsp18* in cSCC spheroids treated with or without ROS scavenger N-Acetyl-L-cysteine (NAC). (F) Percentage of SCC cell viability grown in 2D and 3D after treatment with MMP13 inhibitor WAY 170523 for 72 hours, normalized to control treated with DMSO. Data obtained from n=3 (mean ± S. D.). p-values were obtained from one-way ANOVA test followed by Tukey post-hoc test (A, C, E, F) and from two-tailed *t*-test (B, D); ****p < 0.0001, ***p < 0.001, **p < 0.01 and *p < 0.05.

**
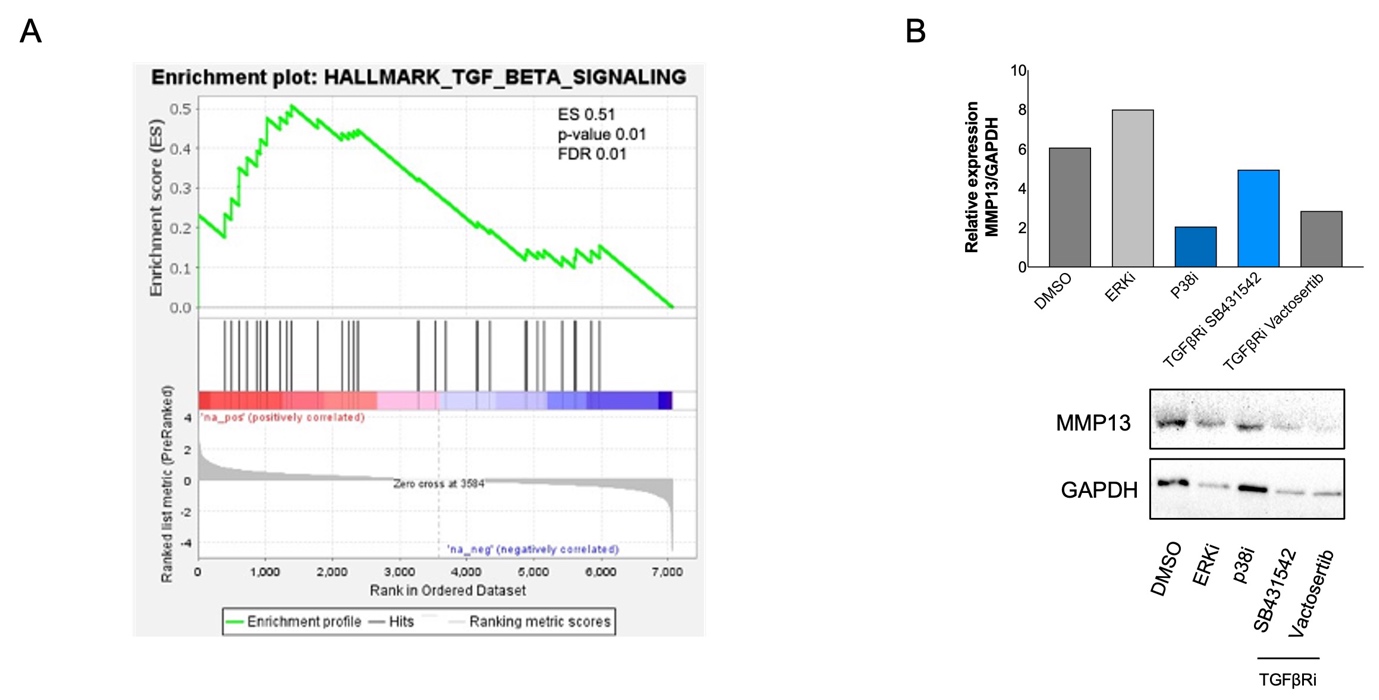
**

**Supplementary Figure 6.** (A) GSEA enrichment analysis of curated mouse gene sets for Kin-1 -/- compared to Kin1-WT. Data from n=3 (mean ± S. D.). (B) Expression of MMP13 in Kin1-/- cells grown as spheroids and treated with the specified inhibitors for 48 hours. Data from an independent experiment. ES: Enrichment score. FDR: False Discovery Rate.

**
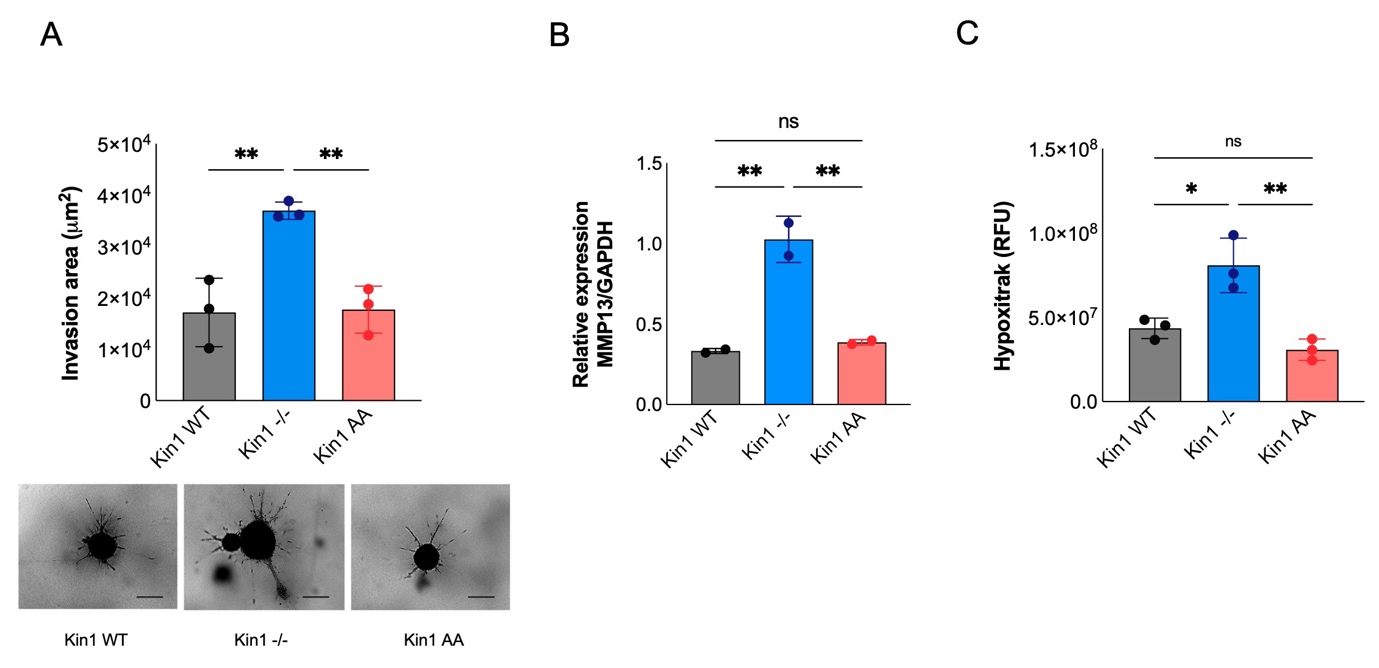
**

**Supplementary Figure 7. Characterisation of Kin1-AA cells.** (A) cSCC spheroid invasion in collagen type I, with representative images after 7 days (bottom panel) (n=3). Scale bar = 400 µm. (B) Expression of MMP13 relative to GAPDH in SCC cell lines assessed by Western blot (n=2). (C) Quantification of HypoxiTRAK™ staining in cSCC spheroids after 72 hours (n=3). p-values were obtained from one-way ANOVA test followed by Tukey post-hoc test; **p < 0.01 and *p < 0.05, ns=not significant.
